# Novel Interactions between FOXM1 and CDC25A Regulate the Cell Cycle

**DOI:** 10.1371/journal.pone.0051277

**Published:** 2012-12-11

**Authors:** Con Sullivan, Youhong Liu, Jingjing Shen, Adam Curtis, Christina Newman, Janet M. Hock, Xiong Li

**Affiliations:** 1 Maine Institute for Human Genetics and Health, Brewer, Maine, United States of America; 2 Center for Molecular Medicine, Xiangya Hospital, Central South University, Changsha, Hunan Province, People's Republic of China; 3 The University of Maine, Orono, Maine, United States of America; Instituto Butantan, Laboratório Especial de Toxinologia Aplicada, Brazil

## Abstract

FOXM1 is a critical regulator of the G1/S and G2/M cell cycle transitions, as well as of the mitotic spindle assembly. Previous studies have suggested that FOXM1 regulates *CDC25A* gene transcription, but the mechanism remains unknown. Here, we provide evidence that FOXM1 directly regulates *CDC25A* gene transcription via direct promoter binding and indirect activation of E2F-dependent pathways. Prior literature reported that CDC25B and CDC25C activate CDK1/cyclinB complexes in order to enable phosphorylation of FOXM1. It was unknown if CDC25A functions in a similar manner. We report that FOXM1 transcriptional activity is synergistically enhanced when co-expressed with CDC25A. The increase is dependent upon CDK1 phosphorylation of FOXM1 at T600, T611 and T620 residues. We also report a novel protein interaction between FOXM1 and CDC25A via the C-terminus of FOXM1. We demonstrate that the phosphorylation of Thr 600 and Thr 611 residues of FOXM1 enhanced this interaction, and that the interaction is dependent upon CDC25A phosphatase activity. Our work provides novel insight into the underlying mechanisms by which FOXM1 controls the cell cycle through its association with CDC25A.

## Introduction

Cell cycle regulation and oncogenesis are inextricably linked through their use of common signaling pathways. The cell cycle relies upon tightly regulated checkpoints at the G1/S and G2/M transitions and fidelity through mitotic spindle formation to ensure cellular integrity. Progression through the cell cycle relies upon a complex temporal interplay among assorted cyclins, associated cyclin-dependent kinases (CDKs), and CDK inhibitors [1]. Cyclins, CDKs, and CDK inhibitors require precise regulation at the DNA and protein levels in order to fulfill these integral functions.

The *Forkhead box M1* (*FOXM1*) gene belongs to the evolutionarily-conserved FOX superfamily of transcriptional regulators. The human FOXM1 is spliced into three classes of transcripts FOXM1A, FOXM1B and FOXM1C, [2]. Recent studies revealed critical roles for FOXM1 in mediating G1/S and G2/M cell cycle transitions, chromosome segregation, and cytokinesis [3]–[6]. Phosphorylation at the C-terminal region is required for the transcriptional activation of FOXM1 during different stages of the cell cycle [7]. FOXM1 contains two RXL docking sequences at the N-terminus, which are bound by CDK2/cyclin E during G1 and S phase and CDK1/cyclin B during G2 phase. In addition, the transcriptional activation of FOXM1 requires an LXL motif for binding of CDK/cyclin complexes, for efficient phosphorylation of FOXM1B Thr residue 596 that is essential for the recruitment of co-transactivator p300/CBP [7]. Raf/MEK/MAPK mediates the phosphorylation of FOXM1 on residues S331 and S704 in late S phase and stimulates nuclear translocation [8]. Moreover, phosphorylation of residues T600 and T611 is required for relief of the inhibitory function of the N-terminal repressor domain [9]. After activation, FOXM1 regulates genes important for cell cycle progression. FOXM1 promotes G1/S transition through its relief of p21^Cip1^ and p27^Kip1^ inhibition [10] and its regulation of *JNK1* [11]. It has also been implicated, although not directly shown, to mediate the transcriptional regulation of the dual phosphatase gene *cell division cycle 25A (CDC25A)* [6], [10], [12].

To facilitate G2/M transition, FOXM1 is activated by PLK1-mediated phosphorylation (S715 and S724), which in turn upregulates *PLK1* [5], [13]. Activated FOXM1 can trigger *cyclin B* transcription through direct promoter binding, thereby exerting potent effects on mitotic entry [14]–[16]. Additionally, FOXM1 regulates the transcription of *cyclin A* [15], [17], *AURKB* [5], and *CDC25B* [6]. FOXM1-deficient cells exhibit chromosomal instability, which has been attributed to its role in mediating the transcription of genes critical to proper chromosome segregation and cytokinesis [14], [18]. FOXM1 mediates the transcription of *CENPF*, which is essential to kinetochore localization and the spindle assembly checkpoint [14]. Additionally, FOXM1 regulates the transcription of mitotic spindle assembly checkpoint genes such as *CENPA*, *NEK2*, and *KIF20A* [10], [19].

CDC25 phosphatases catalyze cell cycle progression by dephosphorylating and activating the cyclin-dependent kinase (CDK) complexes, which in turn regulate cell cycle progression. Three CDC25 isoforms have been identified in mammalian cells: CDC25A, CDC25B and CDC25C. Each regulates different phases of the cell cycle [20]–[22]. CDC25A mainly regulates G1/S cell cycle transition and S phase progression by activating CDK2/cyclin E and CDK2/cyclin A complexes [23], [24]; it also plays a role in the G2/M cell cycle transition by activating CDK1/cyclin B complexes [25], [26].

A positive feedback model between FOXM1 and CDC25B has been proposed in which CDC25B phosphatase is a direct target for FOXM1 transcriptional activation [6], CDC25B protein activates CDK2/cyclin E or CDK1/cyclin B complexes through dephosphorylation, and the activation of CDK/cyclin complexes maintains phosphorylation of FOXM1 [7]. We hypothesize that a similar positive feedback loop of signal transduction may exist between FOXM1 and CDC25A. We previously reported that low doses of arsenic promoted cell proliferation and G1/S cell cycle progression in the human mammary epithelial cell line MCF10A; we used high throughput methods to identify upregulation of CDC25A twenty four hours after low doses of arsenic exposure [27]. We observed that upon long-term exposure to low doses of arsenic, there is a consistently elevated level of FOXM1 and CDC25A expression (unpublished data). These data suggested that there were functional and structural interactions between FOXM1 and CDC25A. However, such interactions between FOXM1 and CDC25A have not been previously characterized.

In this study, we show, for the first time, that FOXM1 directly binds at specific *cis*-regulatory consensus sites and activates the *CDC25A* promoter. We also report that FOXM1 indirectly activates the *CDC25A* promoter through an E2F-dependent mechanism. Additionally, FOXM1 transcriptional activity is synergistically enhanced when co-expressed with CDC25A. Consistent with known mechanisms involving CDC25B and CDC25C, our data support a CDC25A-CDK1-FOXM1 signal transduction pathway that promotes the transcriptional activity of FOXM1. Our data also support a new mechanism in which FOXM1 and CDC25A proteins interact via the C-terminus of FOXM1. The phosphorylation of Thr 600 and Thr 611 residues of the FOXM1 protein enhanced the interaction and the interaction required a functional CDC25A with intact phosphatase activity. This study reveals novel transcriptional and protein-protein interaction mechanisms involving FOXM1 and CDC25A that influence how cell cycle progression is regulated.

## Materials and Methods

### Cell culture

Human U2OS osteosarcoma and HEK293T cells (American Type Culture Collection, ATCC, Manassas, VA) were cultured at 37°C, 5% CO_2_ in Dulbecco's Modified Eagle Medium (DMEM) supplemented with 10% fetal bovine serum (FBS) and 1% penicillin/streptomycin (P/S). CWR22rv prostate cancer cells (obtained from Dr. Chinghai Kao, Department of Urology, Indiana University School of Medicine [28]) were maintained in RPMI1640 supplemented with 10% FBS and 1% P/S.

### Antibodies

Western blotting was performed with primary antibodies directed against FOXM1, CDC25A, CDK2, FLAG, and β-Actin, which were purchased from Santa Cruz Biotechnology (Santa Cruz, CA). Antibodies against CDK1, CDK4 and CDK6 were purchased from Cell Signaling Technology (Danvers, MA), Anti-MYC antibody was purchased from Invitrogen (Carlsbad, CA). Anti-CDC25A agarose used for immunoprecipitation was purchased from Abcam (Cambridge, MA).

### Plasmids

The primers used to generate these constructs are listed in Table S1. pCMV-XL5-FOXM1B plasmid and pCMV-XL5 control vector were purchased from Origene (Rockville, MD). FOXM1 was amplified with Phusion polymerase (New England Biolabs, MA) and subcloned in the pACT and pBIND vectors at their *BamHI* and *XbaI* sites (Promega, Madison, WI) and the p3×FLAG-CMV-14 (Sigma) vector at the *HindIII* and *XbaI*. A series of deletion constructs were made by PCR with Phusion polymerase. All FOXM1 deletion constructs were subcloned in pACT and pBIND at the *BamHI* and *XbaI* sites. The N-terminal deletion (ΔN) covered amino acids 236–763. The C-terminal fragment covered amino acids 330–763. The C-terminal deletion (ΔC) covered amino acids 1–329. The N-terminal fragment incorporated amino acids 1–235. The WHD deletion (ΔWHD) included a fusion of amino acids 1–235 and 330–736. This was accomplished using a PCR sewing technique previously described [29]. The WHD fragment incorporates amino acids 236–329.

The CDC25A plasmid was purchased from Origene and subcloned in-frame in the pACT expression vector at the *BamHI* and *XbaI* restriction sites or the pCMV3Tag9 (Agilent, La Jolla, CA) expression vector at the *BamHI* and *XhoI* restriction sites. The site-directed, phosphatase-dead mutant C431S was created using the QuikChange II site-directed mutagenesis kit according to the manufacturer's recommendations. The FOXM1 binding site reporter plasmid, 6×FOXM1-luc, was generated by annealing two primers containing TACGTTGTTATTTGTTTTTTTCG repeated six times and ligating into the pGL3-basic vector. A 2210 bp fragment of the *CDC25A* promoter and 5′UTR (−1962 through +248) was amplified by PCR from genomic DNA derived from U2OS cells using Phusion polymerase and subcloned in pGL3-Basic (Promega) at the *BglII* and *HindIII* restriction sites. Deletion constructs were subcloned in pGL-3 Basic at the *BglII* and *HindIII* restriction sites using Phusion polymerase: a 1413 bp fragment (−1962 through −549) and a 224 bp fragment containing three putative FOXM1 binding sites (−1302 through −1078). A 583 bp CDC25A promoter fragment (−548 through +34) was a generous gift from Dr. Daniel DiMaio (Yale University Cancer Center, New Haven, CT). QuikChange II site-directed mutagenesis (Agilent) was performed to alter the three putative FOXM1 binding sites and the two E2F binding sites, according to the manufacturer's instructions (Agilent).

### Cell cycle

U2OS cells were seeded at 1×10^6^ cells per 100 mm dish in DMEM+10% FBS. The following day, the cell culture media was replaced with DMEM+10% FBS+100 ng • ml^−1^ nocodazole (Sigma, St. Louis, MO), and the cells were exposed for 16 h to synchronize their cell cycle in G2/M. At 16 h, cells were washed with 1× phosphate-buffered saline (PBS) and returned to DMEM+10% FBS. At 1, 3, 6, 12, and 18 hours following the re-introduction of DMEM+10% FBS, cells were trypsinized and collected for DNA content analysis by FACS and western blotting.

For FACS, trypsinized cells were collected in 1× PBS and resuspended in 70% ethanol to fix overnight at −20°C. Cells were pelleted, washed twice in 3% BSA in 1× PBS, and pelleted. Cells were resuspended and incubated for 30 minutes at room temperature in propidium iodide staining buffer containing 3% BSA, 40 µg • ml^−1^ propidium iodide, and 0.2 mg• ml^−1^ RNase in 1× PBS. DNA content analyses were carried out using the Accuri C6 flow cytometer (Ann Arbor, MI). Data were analyzed with ModFit (Verity Software House, Topsham, ME).

For western blotting, trypsinized cells were washed in 1× PBS, pelleted, and lysed in MPER+protease and phosphatase inhibitors (Thermo Fisher Scientific, Rockford, IL). Soluble fractions were separated by gel electrophoresis on 4–20% polyacrylamide gels (Bio-Rad, Hercules, CA), transferred to PVDF membranes, and subjected to western blotting with antibodies against FOXM1, CDC25A, CDK2, CDK1, and β-Actin.

### ChIP-PCR

ChIP was performed using Chromatrap Pro-A Spin Columns from the Porvair Filtration Group Ltd. (Ashland, VA). Cells were fixed in 1% formaldehyde for 10 minutes. Fixation reactions were quenched with 0.67 M glycine. U2OS and CWR22rv1 cells were lysed and subjected to sonication to achieve 100–500 bp fragments. For immunoprecipitation of formaldehyde cross-linked chromatin-protein complexes, antibodies against FOXM1 or rabbit IgG (Santa Cruz Biotechnology) were used, and the same amount of chromatin without antibody incubation was used as an input control. The samples were incubated with end-over-end rotation for 3 hours at 4°C. DNA was eluted in the Chromatrap Pro-A spin columns according to the manufacturer's recommendations. DNA was analyzed via semi-quantitative PCR and quantitative RT-PCR. Primers were designed to span the FOXM1 binding sequence of the *CDC25A* promoter, along with appropriate controls. The binding activity of FOXM1 to CDC25A promoter was evaluated by the fold enrichment method. (http://www.invitrogen.com/site/us/en/home/Products-and-Services/Applications/epigenetics-noncoding-rna-research/Chromatin-Remodeling/Chromatin-Immunoprecipitation-ChIP/chip-analysis.html)

### FOXM1 overexpression and FOXM1 siRNA knockdown

U2OS cells were plated at 1×10^6^ cells per 60 mm dish. For overexpression, the next day, cells were transfected with pCMV-XL6-FOXM1 or pCMV-XL-6 using Lipofectamine 2000 according to the manufacturer's recommendations. For siRNA knockdown, cells were transfected with ON-TARGET Plus siRNA pools targeting FOXM1 (Dharmacon, Lafayette, CO) using the DharmaFECT reagent, according to the manufacturer's recommendations (Dharmacon). At 48 h, cells were washed with 1× PBS and lysed in MPER+1× protease and phosphatase inhibitors (Thermo Fisher Scientific). Soluble fractions were separated by PAGE on 4–20% gels, transferred to PVDF membranes, and subjected to western blotting with antibodies against FOXM1, CDC25A, and β-Actin.

### Dual luciferase reporter assays and western blot

U2OS cells were plated in 24 well dishes at 1×10^5^ cells per well. The next day, cells were co-transfected with indicated plasmids (total amount of 800 ng) using Lipofectamine 2000 transfection reagents, according to the manufacturer's recommendations. Ten nanograms of the pRL-SV40 plasmid were used for normalization. After incubation for 48 h, cells were washed with 1× PBS and lysed with 1× Passive Lysis Buffer (Promega) and subjected to luciferase reporter assays. Luciferase reporter assays were performed using the Dual Luciferase Assay kit (Promega). Data were collected using a GloMax single tube luminometer (Promega). All firefly luciferase outputs were normalized to *Renilla* luciferase activities.

To identify the CDK substrates involved in CDC25A-activated FOXM1 transcriptional activity, ON-TARGET siRNA targeting CDK1, CDK2, CDK4 or CDK6 (Dharmacon) were co-transfected with the indicated plasmids. After incubation for 48 h, cells were washed with 1× PBS and lysed in MPER+protease and phosphatase inhibitors (Thermo Fisher Scientific). The FOXM1 transcriptional activity was tested by dual luciferase assay. Soluble fractions were separated by PAGE on 4–20% gels, transferred to PVDF membranes, and subjected to western blotting with antibodies against CDK1, CDK2, CDK4 and CDK6, and β-Actin was used as loading control.

### Mammalian two-hybrid system

Mammalian two-hybrid experiments were performed using the Checkmate system, according to the manufacturer's recommendations (Promega). Briefly, U2OS cells were co-transfected with 300 ng of the bait plasmid pBIND-FOXM1, which contains the yeast GAL4 DNA-binding domain fused in-frame with sequence encoding FOXM1 (or the indicated deletion construct), 300 ng of the prey plasmid pACT-CDC25A, which contains the HSV VP16 activation domain fused in-frame with sequence encoding CDC25A (WT or C431S), and 200 ng of the reporter plasmid pG5-luc, which contains five GAL4 binding sites upstream of a minimal TATA box and the firefly luciferase reporter gene. The pBIND plasmid and its derivatives, also express *Renilla* luciferase, which was used for normalization. After incubation for 2 days, cells were washed with 1× PBS, lysed with 1× Passive Lysis Buffer (Promega) and subjected to luciferase reporter assays. Luciferase reporter assays were performed using the Dual Luciferase Assay kit (Promega). Data were collected using the GloMax single tube luminometer (Promega). All firefly luciferase outputs were normalized to *Renilla* luciferase activities.

### Co-immunoprecipitation

Approximately 1×10^6^ 293T cells were plated in 60 mm dishes so that they were ∼90% confluent on the day of transfection. 293T cells were co-transfected with 4 µg of p3×FLAG-CMV-14-FOXM1 (or control p3×FLAG-CMV-14) and 4 µg of pCMV-3Tag9-CDC25A (or control pCMV-3Tag9) using Lipofectamine 2000 transfection reagent, according to the manufacturer's recommendations. After 48 hours, cells were washed in 1× PBS and lysed in M-PER containing protease and phosphatase inhibitors. Soluble fractions were incubated with anti-FLAG M2 antibody resin (Sigma) overnight at 4°C with end-over-end mixing. The resin was washed three times in BupH TBS, pH 7.2 (Thermo Fisher Scientific) containing 0.1% Tween 20 and eluted with 3× FLAG peptide (Sigma). The eluted protein complex was then analyzed by Western blotting.

For the detection of the natural protein interactions of FOXM1 and CDC25A, 6×10^6^ U2OS cells were lysed in MPER buffer containing 1× protease/phosphatase inhibitors (Thermo Fisher Scientific). Whole cell lysates were pre-cleared with protein G sepharose (Sigma Aldrich) and normal rabbit IgG overnight at 4°C with end-over-end mixing. Cell lysates were then incubated with anti-CDC25A antibody (Santa Cruz) overnight at 4°C and then with protein G sepharose for 1 h at 4°C. The resin was washed three times, and the eluted protein complex was analyzed by Western blotting using the anti-FOXM1 antibody (Santa Cruz).

Approximately 1×10^6^ U2OS cells were plated in 60 mm dishes so that they were ∼90% confluent on the day of transfection. U2OS cells were transfected with 8 µg of p3×FLAG-CMV-14-FOXM1 (wild-type), FOXM1(T600A), FOXM1(T620A) and FOXM1(L656A), using Lipofectamine 2000 transfection reagent, according to the manufacturer's recommendations. After 48 hours, cell lysates were coimmunoprecipitated with anti-CDC25A antibody agarose, followed by Western blot with monoclonal antibody against FLAG tag.

### Statistics

All statistics were performed with GraphPad Prism 5.01. The asterisks in each figure indicate statistically significant changes with *P* values calculated by the indicated test: *, *P*<0.05; **, *P*≤0.01; and ***, *P*≤0.001. *, *P*<0.05 was considered statistically significant. ^Δ^, *P*>0.05 represented no significant difference.

## Results

### FOXM1 and CDC25A exhibit coordinated protein expression with cell cycle progression

**Figure 1:**
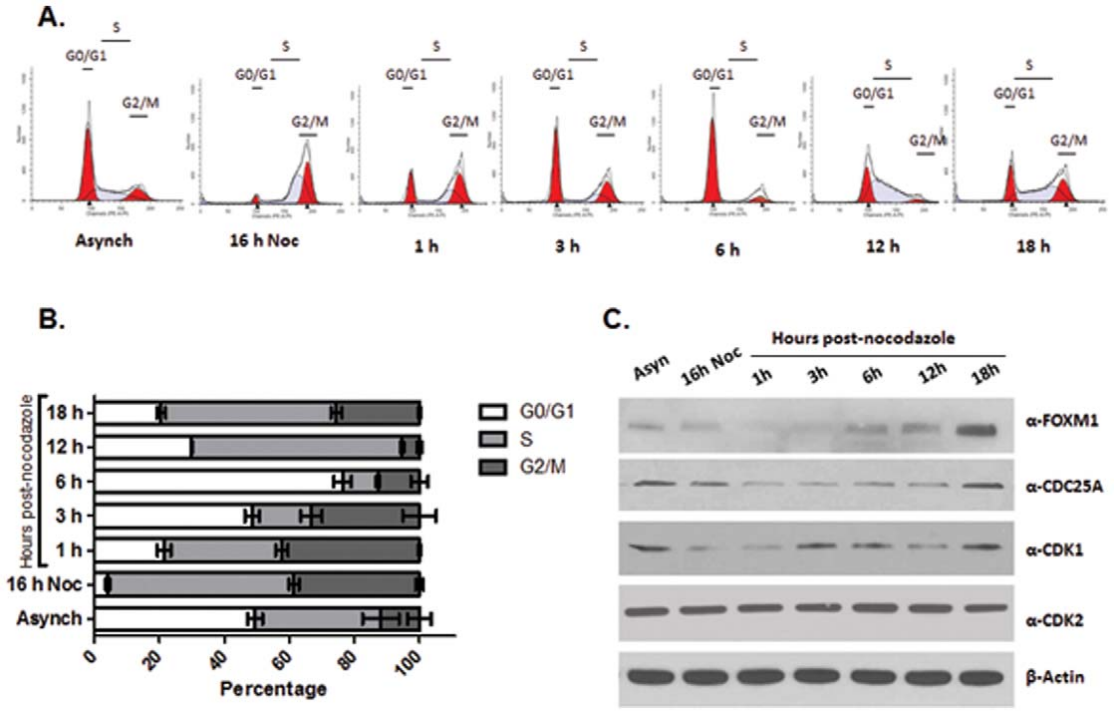
Coordinated expression of FOXM1 and CDC25A during the cell cycle progression. (A) Representative flow cytometry analysis shows cell cycle progression in U2OS cells. U2OS were synchronized with nocodazole for 16 h and then stimulated to re-enter the cell cycle by addition of medium containing 10% fetal bovine serum. Asynchronous cells (Asynch) were included as controls. Cell cycle was analyzed by flow cytometry at release (16 h Noc) and at 1, 3, 6, 12, and 18 h after release. (B) Mean percentage (± SD) of cells in each phase of the cell cycle following 16 h nocodazole treatment and after 1, 3, 6, 12, 18 h release (N = 3). Asynchronous (Asynch) cells were included as controls. The analysis indicates that the cells at early, middle and late G1 phase are at 1, 3 and 6 h respectively, S phase is maximal at 12 h while G2/M phase occurs at 16 h Noc and 18 h after release. (C) Cells at the continuous cell cycle phases were collected and processed for western blotting analysis with antibodies against FOXM1, CDC25A, CDK1 and CDK2. FOXM1 expression increased as cells progressed from G1 through S and into G2/M, and degraded when cells exited to G2/M (1 h). CDC25A and CDK1 exhibited a similar expression profile. CDK2 expression levels were relatively constant throughout the cell cycle. β-Actin expression was used as a loading control.

FOXM1 and CDC25A are known as critical regulators of the G1/S and G2/M transitions during the cell cycle [1], [10], [11], [30]. CDC25A phosphatase activates CDK/cyclin complexes, which then phosphorylate downstream targets and promotes cell cycle progression [31]. CDC25A primarily activates CDK2/cyclin E and CDK2/cyclin A complexes during the G1/S transition [23], [24], and CDK1/cyclin B complex during the G2/M transition and mitosis [25], [26]. We sought to detect the expression of FOXM1, CDC25A, CDK1 and CDK2 during cell cycle progression in the U2OS cell model. U2OS cells were synchronized by treatment with nocodazole (100 ng • ml^−1^) for 16 h. They were then released into growth media containing 10% FBS so that cells were stimulated to re-enter the cell cycle. After treatment with nocodazole for 16 h, most cells were synchronized in both S phase (31.1%) and G2/M phase (65.3%), and very few cells were at G0/G1 phase (3.6%). At 1 h post-release, cells were exiting M phase, and entering into early G1 phase (∼21.5%). By 3 h post release, approximately 48.6% of the cells were entering G1 phase. At 6 h post release, most cells were at the G0/G1 phase (76.4%). By 12 h post-release, cells were entering S phase (64.8%), and by 18 h post-release, cells were in late S (∼53.9% in S phase) or G2/M phase (25.6% in G2/M) (Fig. 1A).

The analyses indicated that most cells at early, middle and late G1 phase are detected at 1, 3 and 6 h post-release respectively. S phase is maximal at 12 h. Released cells entered G2/M at 18 h (Fig. 1B). We collected cells at each of these time points to ascertain if there was a coordinated expression pattern which might indicate co-regulation during cell cycle progression. Western blotting revealed coordinated protein expression in FOXM1 and CDC25A upon release of cells from nocodazole treatment (Fig. 1C). FOXM1 protein expression increased as cells progressed from G0/G1 (1 h) through S (12 h) and into G2/M phase (18 h), and degraded when cells exited to M phase (1 h). CDC25A exhibited an expression profile over time that was similar to FOXM1. CDK1 expression levels increased at the late G1 or early G2 phases, and CDK2 expression levels were relatively constant throughout the cell cycle. These findings suggested that FOXM1 regulates G1/S and G2/M cell cycle transition through its interactions with CDC25A, a protein that has long been suspected of being regulated by FOXM1 [6], [10], [12].

### FOXM1 transcription factor regulates CDC25A gene expression

**Figure 2:**
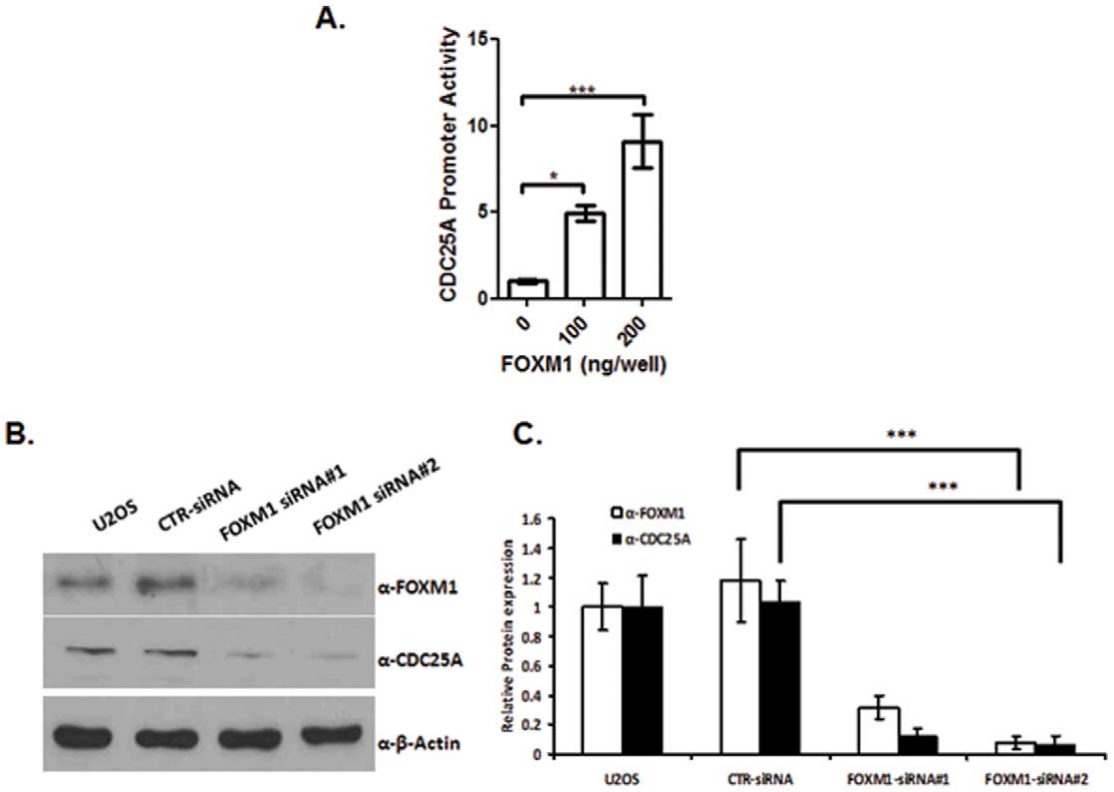
The FOXM1 transcription factor regulates *CDC25A* gene expression. (A) FOXM1 increased the transactivation of *CDC25A* promoter. U2OS cells were co-transfected with pGL3-CDC25A promoter reporter along with increasing doses of pACT-FOXM1 for 48 h. pACT vector was used as the empty vector, and pRL-SV40 expressing *Renilla* luciferase was used as an internal control. Data represent the mean ± SD (N = 3). *, p<0.05; ***, p<0.001. (B) siRNA-mediated silencing of FOXM1 decreased CDC25A expression. U2OS cells were transiently transfected with siRNA pools targeting FOXM1 or non-target control for 48 h. A U2OS non-transfected control was also used. The protein expression of FOXM1 and CDC25A were detected by Western blot. β-Actin expression was used as a loading control. The western blot was repeated three times and the protein expression was analyzed by the Adobe Photoshop CS4 software (Adobe Systems, San Jose, CA). The difference of band density between the FOXM1-siRNA and control siRNA was analyzed by the paired t test. ***, p<0.001.

CDC25A transcription is regulated by E2F and c-MYC transcription factors in a cell cycle-dependent manner (15, 40). Previous reports indicated that FOXM1 regulates *CDC25A* gene expression, but the mechanism by which this occurred was not established [6], [10], [12]. We wanted to directly characterize the role of FOXM1 in activating the *CDC25A* promoter. We used a *CDC25A* promoter-driven luciferase construct containing the promoter and 5′UTR (−1896 through +123) to measure the effect of FOXM1 on *CDC25A* promoter activity. In a co-transfection study initiated in U2OS cells, we observed that increasing levels of FOXM1 protein triggered a dose-dependent increase in CDC25A promoter activity (Fig. 2A). Confirming this regulation, western blotting revealed that siRNA-mediated knockdown of FOXM1 protein expression led to a corresponding decline in CDC25A protein levels (Fig. 2B and 2C). Siomycin A, a small molecule FOXM1 inhibitor, decreased the gene transcription of CDC25A in U2OS cells (See Fig. S1). These data establish a direct role for FOXM1 in the regulation of CDC25A gene expression.

### FOXM1 regulates CDC25A gene transcription through direct binding and through an indirect mechanism involving the E2F pathway

By using the Transcription Elements Search System (TESS) (http://www.cbil.upenn.edu/cgi-bin/tess/tess), we identified three putative FOXM1 consensus sites in the *CDC25A* promoter and 5′UTR (−1896 through +123). The identified FOXM1 putative sites are located at positions −1019 through −1012 (GTAAATAT), −891 through −884 (TTATTTGC), and −875 through −868 (TGTTTGCT). Two E2F binding sites were identified at positions −156 through −149 (TTTGGCGC) and −97 through −90 (CCGCGAAA) at the 3′ end of the *CDC25A* promoter (See Fig. S2) [30], [32].

**Figure 3:**
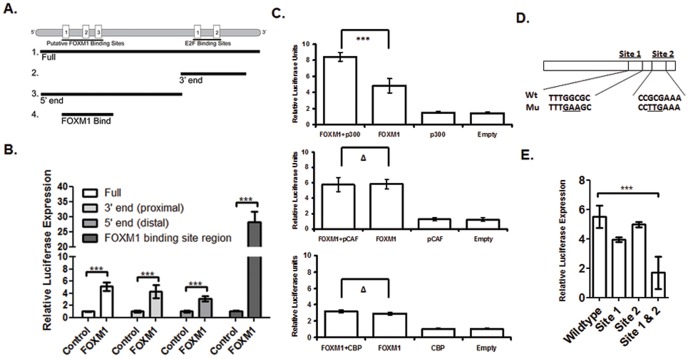
FOXM1 regulates the gene transcription of CDC25A by direct DNA binding as well as the E2F pathway. (A) Schematic diagram of CDC25A promoter showing the 3′ distal end containing two E2F binding sites, the 5′ proximal end including three putative FOXM1 binding sites and excluding E2F binding sites, and a 224 bp fragment containing the three putative FOXM1 binding sites. (B) FOXM1 increased the transactivation of full-length and truncated CDC25A promoter constructs, indicating both direct and indirect FOXM1 induction. U2OS cells were co-transfected with pACT-FOXM1 or pACT empty plasmid and the reporter plasmids containing full length and truncated CDC25A promoters. pRL-SV40, a *Renilla* luciferase report vector, was co-transfected as the control. Data were normalized to *Renilla* luciferase activities and are presented as the mean ± SD (N = 5). ***, p<0.001. (C) FOXM1 recruits p300, but not PCAF or CBP, to activate CDC25A promoter. U2OS cells were co-transfected with pACT-FOXM1 (or pACT control) and pCMV-p300, or pCMV-pCAF, or pCMV-CBP (or empty vector control), along with pGL3-CDC25A promoter for 48 h. pRL-SV40 was co-transfected as the normalizing control. Data are normalized to *Renilla* activity, and represented as the mean ± SD. The difference was analyzed by paired t test. ***p<0.001, Δ>0.05. (D) Representation of the wild-type and mutated E2F consensus binding sequences at CDC25A promoter. (E) Two E2F binding sites are required for FOXM1-activated CDC25A promoter transactivation. U2OS cells were co-transfected with pACT-FOXM1 or empty vector control and the reporter plasmids containing the indicated CDC25A promoters (wild-type or mutated E2F binding sites). Data are presented as the mean fold induction of FOXM1 activity over control ± SD. *** p<0.001.

To investigate the roles of individual FOXM1 and E2F binding sites in FOXM1-induced transactivation of CDC25A promoter, we generated targeted deletions of the *CDC25A* promoter to identify regions of enhanced FOXM1 responsiveness (Fig. 3A). The 3′ end CDC25A promoter construct contains two E2F binding sites, and the 5′ end CDC25A promoter construct contains the three putative FOXM1 binding sites, and excludes the E2F binding sites. We also created a construct that contained sequence solely in the region of the three putative FOXM1 binding sites. The data from the co-transfection experiment showed that the deletion containing the 5′ end only (with FOXM1 binding sites) exhibited a 4-fold FOXM1-dependent activation. FOXM1 strongly activated the promoter construct containing the FOXM1 binding site region alone by 29-fold, confirming the biological function of FOXM1 binding sites (Fig. 3B).

To clarify how the promoter containing only the three putative FOXM1 binding sites is able to drive gene transcription in the absence of the minimum promoter, we tested if the co-activators p300, CBP, and pCAF coordinated with FOXM1 activated the transcriptional activity of the 5′ end CDC25A promoter. Co-activators may directly activate basal transcriptional machinery to recruit RNA Pol II and promote formation of stable transcription pre-initiation complexes. Co-activators may also alter DNA directly by acetylating and activating transcription factors and chromatin associated proteins. Histone function can be modified by co-activators via acetylation of nucleosomal histones to modify the functioning of promoters of activated genes. Co-activators are themselves part of an active proteomic network as, once activated, they may link to other transcriptional co-activators to expand the diversity of genes activated [33]. The 5′ end CDC25A promoter construct was co-transfected with plasmids containing FOXM1, with or without p300, CBP and pCAF. In contrast to the control vector plus FOXM1, p300, but not CBP and pCAF, significantly increased FOXM1-induced CDC25A transcriptional activity (Fig. 3C).

The 3′ end of the *CDC25A* promoter, which is devoid of FOXM1 binding sites, still exhibited luciferase activity upon FOXM1 overexpression (3.8-fold, Fig. 3B), suggesting an additional mechanism. *CDC25A* is known to be transcriptionally regulated by the E2F pathway, possessing two E2F binding sites within the 3′ end of the *CDC25A* promoter [30], [32]. It is not known if FOXM1 regulates *CDC25A* transcription through an E2F-dependent mechanism. We found that knockdown of FOXM1 significantly decreased the levels of E2F1 and E2F2 transcripts, but not E2F3 in U2OS cells (E2F3 data not shown, See Fig. S3). We performed site-directed mutagenesis of the *CDC25A* promoter to alter both E2F binding sites (Fig. 3D). In U2OS cells, mutation of both E2F sites led to a significant reduction of *CDC25A* promoter-luciferase activity upon ectopic FOXM1 expression (Fig. 3E). Taken together, the data indicate that FOXM1-induced *CDC25A* gene transcription is induced both by direct binding and by an indirect mechanism involving the E2F pathway.

### FOXM1 protein physically interacts with the CDC25A promoter

**Figure 4:**
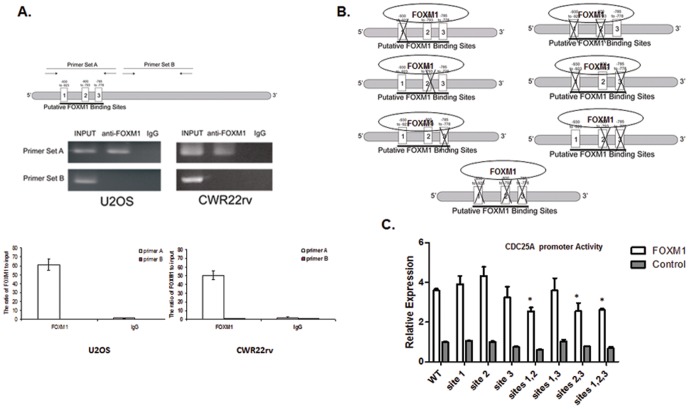
FOXM1 directly binds to the putative FOXM1 consensus binding sequences located on the CDC25A promoter. (A) FOXM1 binding to CDC25A promoter was confirmed by ChIP-qPCR. Primer set A was designed to span three FOXM1 binding sites, and primer set B, targeting a region of the promoter that did not include the FOXM1 binding sites, was used as the control. Chromatin immunoprecipitations of U2OS cells were prepared using anti-FOXM1 antibody. Semi-quantitative PCR was performed using the primer sets A and B, and the PCR products were detected by electrophoresis (Top). Quantitative PCR was performed using the primer sets A and B, and the binding activity of FOXM1 to CDC25A promoter was evaluated by the fold enrichment method. (B–C) The three FOXM1 binding sites on the CDC25A promoter are functionally redundant (B) Schematic diagram showing the mutations of the three FOXM1 binding sites on the CDC25A promoter. (C) U2OS cells were transfected with pACT-FOXM1 or empty vector control, along with the indicated CDC25A promoter-luciferase reporter construct. Targeted mutation of single FOXM1 binding sites did not significantly affect CDC25A transactivation. Mutations of sites 1 and 2, sites 2 and 3, and sites 1, 2, and 3 significantly reduced the transactivation of the CDC25A-luciferase reporter. Data were normalized to *Renilla* luciferase activities and are presented as the mean wild-typefold induction over empty vector control ± SD (N = 3). Data were subjected to one-way ANOVA (significance level α = 0.05) and Dunnett's multi-comparison post-hoc tests (mutations vs. wild-type). * P<0.05.

The potential protein-DNA binding of FOXM1 at CDC25A promoter was tested by the chromatin immunoprecipitation (ChIP)- quantitative PCR assay. Anti-FOXM1 antibody was used to immunoprecipitate regions of chromatin bound by FOXM1 in U2OS osteosarcoma cells and CWR22rv prostate cancer cells. The immunoprecipitated chromatin was used in semi-quantitative PCR and real time quantitative PCR to test for direct binding of FOXM1 to CDC25A promoter. Primers were designed to amplify the region of the CDC25A promoter that contained the three putative FOXM1 binding sites (primer set A) or the region lacking the three binding sites (primer set B). In both U2OS and CWR22rv cells, there was robust expression of the FOXM1 amplicon which incorporated the FOXM1 binding sites. There was no amplification of a downstream region of the CDC25A promoter that lacked FOXM1 binding sites, and no amplification was observed in IgG controls (Fig. 4A). These findings confirm that FOXM1 directly binds to the three FOMX1 binding sites found in the CDC25A promoter.

### The FOXM1 binding sites in the CDC25A promoter are functionally redundant

As shown in Fig. 3B, the *CDC25A* promoter deletion containing three FOXM1 binding sites (224 bp FOXM1 portion) exhibited the most robust activation, supporting the idea that FOXM1 directly binds these three sites. The ChIP-qPCR and reporter studies support a direct FOXM1 binding to the *CDC25A* promoter, regulating its transcriptional activity. These three FOXM1 binding sites lie in close proximity to one another in the *CDC25A* promoter. It is unknown if all three binding sites are required for FOXM1-induced CDC25A transcription. We performed site-directed mutagenesis to change one, two or three of the FOXM1 binding sites in the 224 bp FOXM1 portion of the CDC25A promoter, as shown in Fig. 4B. We compared the transactivation of promoter containing the mutated FOXM1 binding sites with the wild-type FOXM1 binding sites when exposed to ectopic FOXM1 expression. In comparison to the truncated promoter containing wild-type FOXM1 binding sites, the mutation of one of the three FOXM1 binding sites did not significantly decrease the transactivation (Fig. 4C), indicating a degree of functional redundancy. When sites 1 and 2, sites 2 and 3, or sites 1, 2, and 3 were mutated, we observed a significant decrease in FOXM1-dependent luciferase activity.

### CDC25A bolsters FOXM1 transcriptional activity through CDK1 pathway

**Figure 5:**
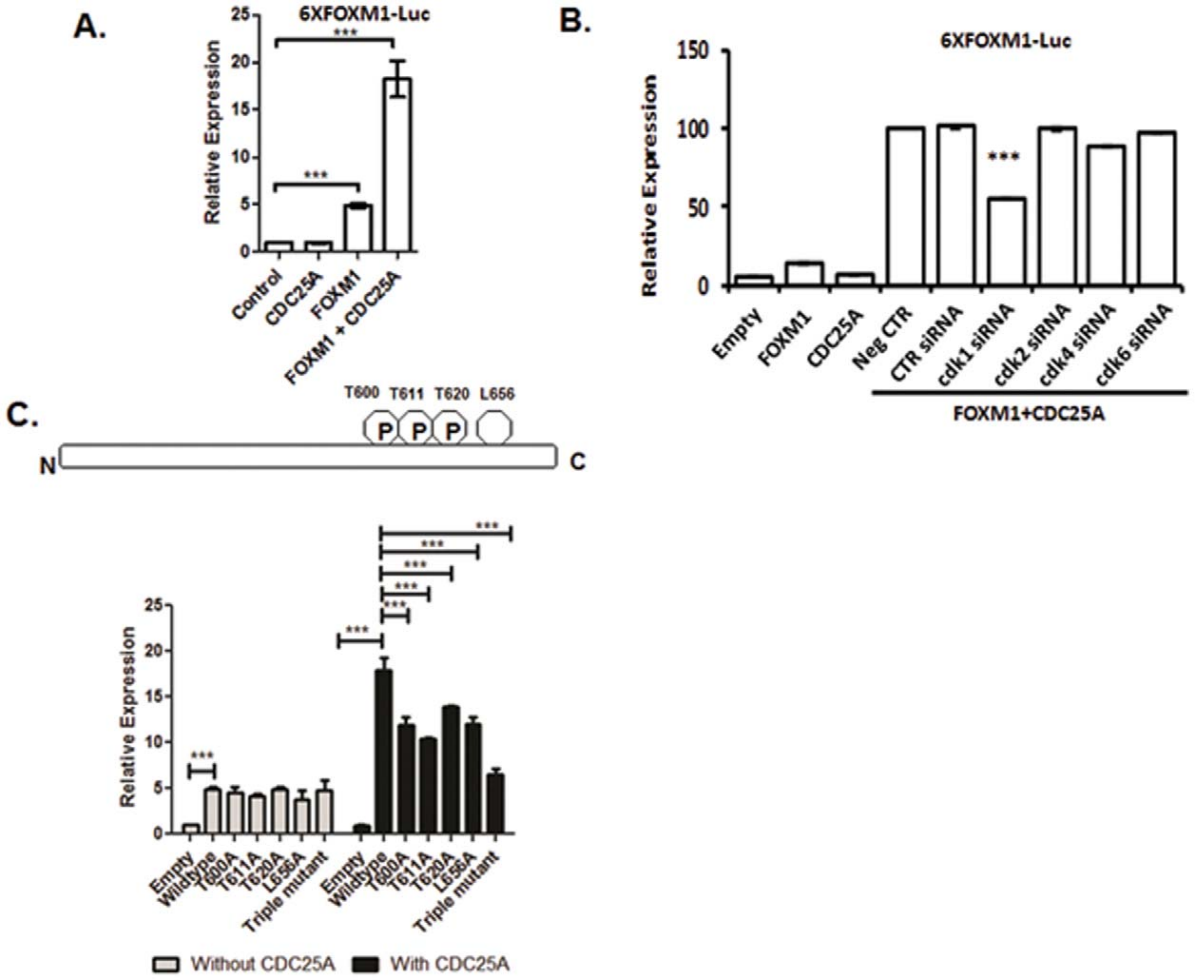
CDC25A regulated FOXM1 transcriptional activity through CDK1-mediated phosphorylation sites. (A) CDC25A activated the transcriptional activity of FOXM1 in U2OS cells. U2OS cells were co-transfected with pACT-FOXM1 or/and pACT-CDC25A, along with the pGL3-6×FOXM1-Luc plasmids containing 6 FOXM1 DNA binding sequences, and pRL-SV40, a *Renilla* luciferase reporter vector was used as the control. Data were normalized to *Renilla* luciferase activities and are presented as the mean ± SD (N = 3). *** p<0.001. (B) siRNA-mediated inhibition of CDK1 blocked CDC25A-mediated FOXM1 transcriptional activity. U2OS cells were co-transfected with pACT-CDC25A, pACT-FOXM1 and pGL3-6×FOXM1-Luc plasmids, or pACT-FOXM1 and pACT-CDC25A together with siRNAs against CDK1, CDK2, CDK4 or CDK6 or control siRNA, and pRL-SV40 was used as the normalizing control. Forty-eight hours after transfection, the cells were lysed, and firefly and *Renilla* luciferase activities were measured. Data were normalized to *Renilla* luciferase activities and are presented as the mean ± SD (N = 3). Data were subjected to one-way ANOVA (significance level α = 0.05) and Dunnett's multi-comparison post-hoc tests (CDK siRNA vs. CTR siRNA). *** p<0.001. (C) CDC25A activated FOXM1 transcriptional activity via the CDK phosphorylation sites T600, T611, and T620 and a LXL docking motif at L656. U2OS cells were co-transfected with pACT-CDC25A and pACT-FOXM1 (wild-type or the mutations of phosphorylation sites/LXL motif), along with pGL3-6×FOXM1-Luc plasmids. pRL-SV40 was used as the normalizing control. Data were normalized to *Renilla* luciferase activities and are presented as the mean ± SD (N = 3). Data were subjected to one-way ANOVA (significance level α = 0.05) and Dunnett's multi-comparison post-hoc tests (mutations vs. wild-type). *** p<0.001.

FOXM1 activation requires interactions with, and phosphorylation by, CDK/cyclin complexes [34]. CDC25B and CDC25C protein activate CDK1/cyclin B complexes by dephosphorylation, allowing the CDK1/cyclin B complex to maintain the phosphorylation of FOXM1 (33). We wanted to test if CDC25A participates in this mechanism. We observed that co-expression of CDC25A and FOXM1 had a synergistic effect on the transcriptional activity of FOXM1 (Fig. 5A). This finding suggests that CDC25A activates FOXM1 transcription.

Because CDC25 phosphatases have been implicated in the control of cell cycle transition by regulating the activities of CDK1 and CDK2, we tested which CDK(s) might play a more critical role in the mechanism of CDC25A-FOXM1 signal transduction. We first used small molecular inhibitors of CDK1 (3-(2-Chloro-3-indolylmethylene)-1,3-dihydroindol-2-one) and CDK2 (inhibitor II) after DNA co-transfection. The IC50 of the CDK1 inhibitor is 5.8 µM and of the CDK2 inhibitor is 60 nM. A 10 µM dose of CDK1 inhibitor is required to significantly block CDC25A-induced FOXM1 transcription activity. This concentration is ∼1.7-fold higher than the IC50 of the CDK1 inhibitor. A 0.4 µM dose of CDK2 inhibitor is required to significantly block CDC25A-enhanced FOXM1 transcription activity. The concentration is ∼6.7-fold higher than the IC50 (See Fig. S4). These data indicate that CDC25A probably enhances FOXM1 transcriptional activity via the CDK1, but not by the CDK2 pathway. The results were validated using siRNA. Consistent with Fig. 5A, co-expression of CDC25A with FOXM1 significantly increased FOXM1 transcriptional activity. This synergistic activation was significantly inhibited when CDK1 expression was knocked down by siRNA. Knockdown of CDK2, CDK4 or CDK6 had no significant effect on the FOXM1-CDC25A activation of a FOXMI-binding site reporter (Fig. 5B). The efficacy of siRNA knockdown was confirmed by western blot (See Fig. S5). These results support a CDC25A-CDK1-FOXM1 signaling pathway.

CDC25B activates the FOXM1 transcriptional activity through the CDK1 phosphorylation of FOXM1 protein, and CDK/cyclin proteins phosphorylate either Ser or Thr residues with a consensus phosphorylation sequence X-S/T-P-X-R/K [7]. We searched for potential CDK phosphorylation sites on the FOXM1 protein using the NetPhos 2.0 server (http://www.cbs.dtu.dk/services/NetPhos/) and identified a series of CDK phosphorylation sites. After screening these phosphorylation sites by site-directed mutagenesis, we identified three phosphorylation sites (T600, T611 and T620) and a second Leu residue within a LXL sequence (L656) involved in the CDC25A-enhanced FOXM1 transcriptional activity. We observed that mutations of these phosphorylation sites and the LXL sequence reduced CDC25A-enhanced FOXM1 transcriptional activity (Fig. 5C). These results support a model in which CDK1/cyclin protein complexes mediate CDC25A-enhanced FOXM1 transcriptional activity via phosphorylation of T600, T611 and T620 residues and the LXL docking motif. [7].

### The C terminus of FOXM1 interacts with CDC25A, and the interaction was enhanced by phosphorylation of T600 and T611 residues

**Figure 6:**
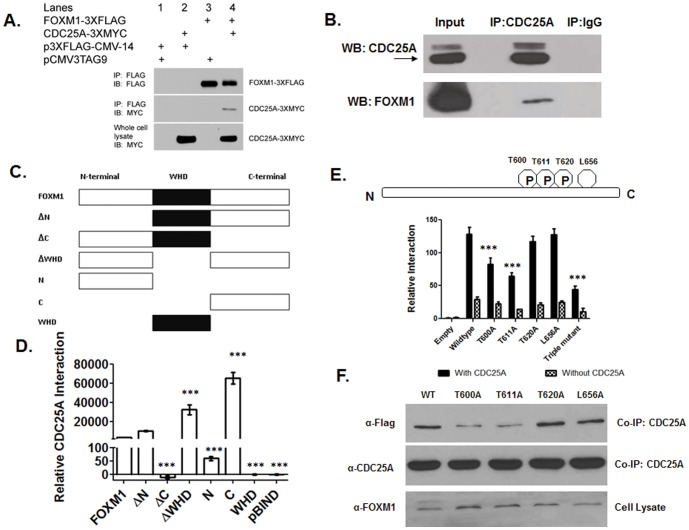
CDC25A phosphatase activates FOXM1 transcriptional activity by direct protein-protein interaction. (A) CDC25A and FOXM1 proteins physically interact. The protein-protein interaction of FOXM1 and CDC25A was confirmed by co-immunoprecipitation. HEK293T cells were co-transfected with p3×FLAG-CMV-14-FOXM1 (FOXM1-3×FLAG) or the empty vector control p3×FLAG-CMV-14, along with pCMV3Tag9-CDC25A (CDC25A-3×MYC) or the empty vector control (pCMV3Tag9). Lysates were immunoprecipitated with anti-FLAG agarose resin, separated by PAGE, and electroblotted to PVDF. Western blot analysis showed that CDC25A-3×MYC co-immunoprecipitates with FOXM1-3×FLAG, supporting the hypothesis that these two expressed proteins interact. (B) The native protein interaction between FOXM1 and CDC25A was detected by co-immunoprecipitation. 6×10^6^ U2OS cells were lysed in MPER buffer containing 1× protease/phosphatase inhibitors. Whole cell lysates were pre-cleared with protein G sepharose and normal rabbit IgG overnight at 4°C with end-over-end mixing. The cell lysates were incubated with anti-CDC25A antibody overnight at 4°C and then with protein G sepharose for 1 h at 4°C. The resin was washed three times, and the eluted protein complex was separated by PAGE and analyzed using the anti-FOXM1 antibody by Western blotting. (C) Schematic diagram showing FOXM1 deletion constructs used to identify FOXM1 domains critical to the interaction with CDC25A. Sequences encoding the specific FOXM1 deletion constructs were subcloned in pBIND. (D) A mammalian two-hybrid assay reveals a critical role for the FOXM1 C-terminus in interactions with CDC25A. pBIND construct (FOXM1, deletion, or empty) was co-transfected with pACT construct (CDC25A or empty) and pG5luc reporter. Data represent the mean ± SD, normalized to *Renilla* luciferase activities (N = 3). Co-expression of the C-terminal FOXM1 and CDC25A revealed a robust interaction. In a construct lacking the C-terminus, the interaction between FOXM1 and CDC25A was significantly diminished. *** p<0.001. (E) Schematic diagram showing the mutations of the CDK/cyclin phosphorylation sites and LXL motif at the C-terminal of FOXM1 protein. pBIND construct (FOXM1 wild-type and FOXM1 with mutations of phosphorylation sites/LXL motifs) was co-transfected with pACT construct (CDC25A or empty) and pGL5-luc reporter. Data represent the mean ± SD, normalized to *Renilla* luciferase activities (N = 3). Data were subjected to one-way ANOVA (significance level α = 0.05) and Dunnett's multi-comparison post-hoc tests (mutations vs. wild-type). *** p<0.001. (F) Mutation of T600 and T611 diminishes FOXM1 protein association with CDC25A by co-immunoprecipitation. U2OS cells were transiently transfected with either FOXM1-3×FLAG construct encoding wild-type FOXM1 (lane 1) or FOXM1 with the following mutations: T600A (lane 2), T611A (lane 3), T620A (lane 4) and L656A (lane 5). U2OS cell lysates were prepared 48 hours after transfection and FOXM1 expression in these cell lysates was detected by Western blot. The same amount of protein was co-immunoprecipitated with anti-CDC25A antibody, and the co-immunoprecipitated proteins were subjected to Western blot with an anti-FLAG antibody. The CDC25A expression in the co-immunoprecipitated proteins was used to test the efficiency of co-immunoprecipitation. The phosphorylation of T600 and T611 enhanced the protein interaction of FOXM1 and CDC25A.

CDC25A regulates cell cycle through interaction with CDK/cyclin complexes [35], including CDK1/cyclin B [36]. Both CDC25A and FOXM1 are thought to interact with CDK1, but a direct protein interaction between CDC25A and FOXM1 has not been characterized previously. We wanted to determine if such a direct protein-protein interaction occurs between FOXM1 and CDC25A. HEK293T cells were co-transfected with plasmids encoding a FOXM1-3× FLAG fusion gene and/or a CDC25A-3×MYC fusion gene. FOXM1-3× FLAG protein was immunoprecipitated from the soluble fraction with an anti-FLAG resin. CDC25A-3×MYC protein was co-immunoprecipitated, supporting a protein-protein interaction with FOXM1-3×FLAG (Fig. 6A).

An endogenous protein-protein interaction of FOXM1 and CDC25A was detectable also in U2OS cells. Following cell lysis, the CDC25A protein complex was captured by protein G sepharose and anti-CDC25A antibody, and FOXM1 protein expression was identified using anti-FOXM1 antibody by western blot. 10% of protein was used as the input (positive control), and IgG was used as negative control. As shown in Fig. 6B, CDC25A and FOXM1 protein expression were high in the CDC25A antibody-captured protein complex, but undetectable in the IgG-captured protein complex. The result indicates that endogenous FOXM1 interacts with CDC25A protein (Fig. 6B).

A mammalian two hybrid assay was performed in U2OS cells to confirm this interaction and determine which FOXM1 domain mediated this interaction (Fig. 6C). GAL4-FOXM1 and VP16-CDC25A exhibited significant interaction relative to control, as measured by normalized luciferase activity, indicating a robust interaction occurs between FOXM1 and CDC25A. FOXM1 proteins, each containing a different domain, were generated by a PCR deletion strategy and expressed as GAL4-FOXM1 deletion mutant proteins. Mammalian two hybrid data collected from these studies support a critical role for the C-terminus of FOXM1 in mediating the FOXM1-CDC25A protein interaction. Neither the N-terminal domain nor the winged helix domain (WHD) of FOXM1 exhibited interactions with CDC25A. In the presence of C-terminal FOXM1, the interaction was >60,000 fold above background. When the C-terminus was deleted, the resultant FOXM1 protein, containing the N-terminus and the WHD, failed to interact with CDC25A (Fig. 6D).

Our findings led us to test the hypothesis that the C-terminal FOXM1 interaction with CDC25A relied upon phosphorylation of residues, which were identified as critical to CDC25A-increased FOXM1 transcriptional activity in Fig. 5C. We further tested if the phosphorylation sites T600, T611 and T620 and the LXL docking motif (L656) enhanced the protein-protein interaction. The mutations of the T600A and T611A, but not the T620A and L656A at the C-terminus, significantly impaired the protein interaction of FOXM1 and CDC25A. The triple mutant T600A/T611A/T620A further impaired the protein interaction of FOXM1 and CDC25A (Fig. 6E). These results were confirmed by co-immunoprecipitation experiments with protein extracts prepared from U2OS transfected with either wild-type FOXM1 or FOXM1 with mutations of phosphorylation sites or the LXL docking motif. These results support the idea that intact phosphorylation of T600 and T611 residues of FOXM1 protein enhanced the protein's interaction with CDC25A (Fig. 6F).

### CDC25A phosphatase activity is required for the CDC25A-activated FOXM1 transcriptional activity, CDC25A and FOXM1 protein interaction and CDK phosphorylation of the FOXM1 protein

**Figure 7:**
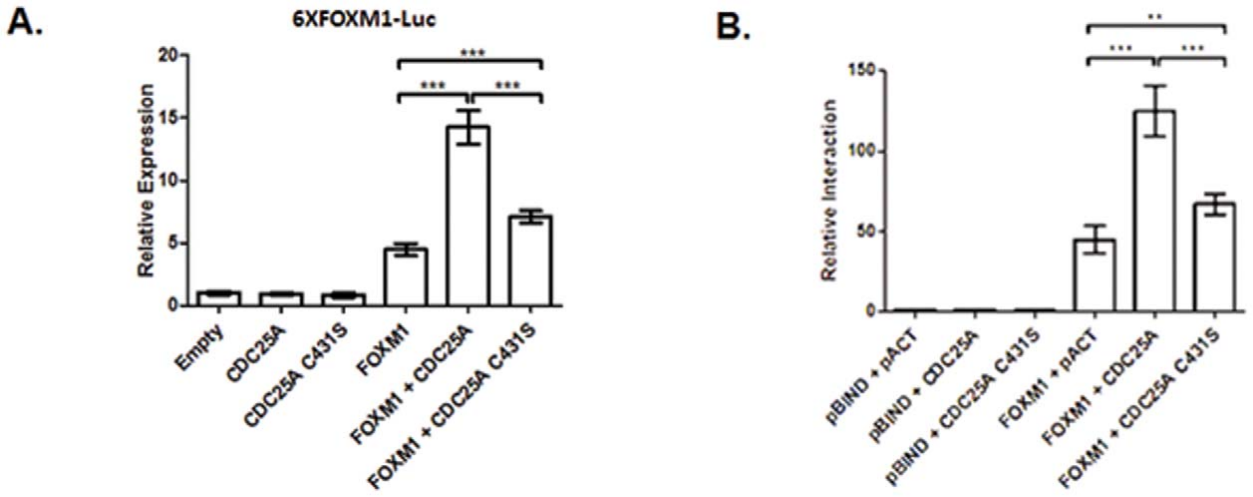
CDC25A phosphatase enzyme activity is required for the CDC25A-activated FOXM1 transcriptional activity and protein-protein interaction between CDC25A and FOXM1. (A) CDC25A phosphatase enzyme activity is required for the CDC25A-activated FOXM1 transcriptional activity. The C431 site in CDC25A protein was changed via site-directed mutagenesis to a serine to create a phosphatase-dead mutant. U2OS cells were co-transfected with pACT-FOXM1 and/or pACT-CDC25A (pACT-CDC25A C431S), along with the pGL3-6×FOXM1-Luc plasmids containing 6 FOXM1 DNA binding sequences. pRL-SV40, a *Renilla* luciferase reporter vector, was used as the control. Data were normalized to *Renilla* luciferase activities and are presented as the mean ± SD (N = 5). *** p<0.001. (B) A mammalian two-hybrid assay confirmed a critical role for CDC25A phosphatase activity in mediating the FOXM1-CDC25A protein-protein interaction. U2OS cells were co-transfected with pBIND-FOXM1 (or pBIND control) and one of following: wild-type pACT-CDC25A, or pACT-CDC25A C431S phosphatase-dead mutant, or pACT control. pG5-luc was used as a normalizing control. Data were normalized to *Renilla* luciferase activities and are presented as the mean ± SD, (N = 5). Loss of phosphatase activity by CDC25A led to a significant reduction in the FOXM1-CDC25A interaction. ** p<0.01; *** p<0.001.

The biological effect of CDC25A phosphatase is potentially dependent on both its abundance and its catalytic activity [37]. CDC25A phosphatase activity is required for dephosphorylation, as well as activation of CDK [38] and the interaction with CDK/cyclin complexes [39]. We investigated if the interaction of FOXM1 and CDC25A is dependent on CDC25A phosphatase activity. Phosphatase activity was destroyed by site-directed mutagenesis of the cysteine at amino acid 431 to serine (C431S) [38]. A mammalian two hybrid assay was used to analyze the interaction when either wild-type CDC25A or mutant CDC25A C431S was co-expressed with FOXM1. We observed that wild type CDC25A had a significantly higher affinity for FOXM1 than the C431S mutant. The data showed that FOXM1-CDC25A protein-protein interaction is dependent upon CDC25A phosphatase activity, as inactivation of CDC25A phosphatase activity significantly decreased the protein-protein interaction (Fig. 7A).

We also investigated if inactivation of CDC25A phosphatase affects FOXM1 transcriptional activity. Wild-type CDC25A or mutant CDC25A C431S was used to test the effect of CDC25A phosphatase activity on FOXM1 transcriptional activity. Consistent with data in Fig. 7A, wild-type CDC25A significantly increased FOXM1 transcriptional activity. However, CDC25A-increased FOXM1 transcriptional activity significantly decreased when CDC25A phosphatase was inactivated by the C431S mutation (Fig. 7 B). CDC25A dephosphorylates and activates CDK/cyclin complexes. The data do not support the idea that FOXM1 is a novel substrate of CDC25A-mediated dephosphorylation. Addition of CDC25A did not decrease, but rather increased the FOXM1 transcriptional activity. The biological effects of the protein interaction of FOXM1 and CDC25A need further investigation. Previous studies reported protein interactions of CDC25A and CDK1 [40] and of FOXM1 and CDK1 [7]. FOXM1 transcriptional activity is dependent on a CDC25A-CDK1-FOXM1 signal transduction pathway, i.e., CDC25A phosphatase activates CDK1, thereby allows the phosphorylation of FOXM1 and transcriptional activation of FOXM1.

## Discussion

It is well known that FOXM1 participates in the G1/S and G2/M cell cycle transition phases, as well as in the assembly of the mitotic spindle [41]. CDC25A is a known G1/S cell cycle regulatory gene [24], [34] that exhibits an expression profile similar to FOXM1, with protein levels steadily increasing through S phase entry and mitosis (Fig. 1C). Transcriptional regulation of *CDC25A* has been shown to be directed by c-MYC in a fibroblast cell line through direct binding of MYC/MAX binding sites [42] and by E2F/Rb pathways [30]. In previous reports, FOXM1 was implicated, although not directly shown, to transcriptionally regulate *CDC25A* transcription [6], [10], [12]. In this study, we identified two novel mechanisms by which FOXM1 regulates *CDC25A* transcription: directly, through interactions with consensus FOXM1 binding sites, and indirectly, through an E2F-dependent mechanism.

We show that with increased FOXM1 protein, there is a concomitant increase in *CDC25A* promoter activity in U2OS cells (Fig. 2A). These findings were supported by data showing that, when FOXM1 was knocked down by siRNA, U2OS cells, exhibited a similar decrease in CDC25A protein expression (Fig. 2B). These data support the hypothesis that CDC25A is a direct target gene of the FOXM1 transcription factor. Through promoter deletion analysis, we identified a 224 bp fragment containing three putative FOXM1 binding sites that exhibited the most robust activation of the reporter (29-fold) (Fig. 3A,and B). These three putative FOXM1 binding sites lie in close proximity to one another on the CDC25A promoter. Their capacity to bind FOXM1 was verified by ChIP-PCR assays (Fig. 4A). Site-directed mutagenesis of FOXM1 binding sites 1 and 2, sites 2 and 3, and sites 1, 2, and 3 each resulted in a significant loss of promoter activity (Fig. 4B, and C). Mutation of a single binding site did not disrupt FOXM1 regulation of the CDC25A promoter. This indicates a level of functional redundancy among the three FOXM1 binding sites. We report for the first time evidence that FOXM1 directly binds to three consensus sites on the CDC25A promoter and provide a good example where, when multiple binding sites for one transcription factor lie in close proximity to one another on a gene promoter, any of the functional binding sites may promote gene transcription [43]. Interestingly, we did not see a complete loss of FOXM1-stimulated promoter activity when all three FOXM1 binding sites in the CDC25A promoter were mutated. We speculate that FOXM1 may indirectly activate the reporter construct by triggering pathways that result in translocation of other transcription factors capable of binding the promoter. Because mutations to the FOXM1 binding sites were made to single nucleotides and not the entire consensus sequence, we speculate that FOXM1, when ectopically expressed, still retains the capacity to bind the mutated consensus sequences, although only in a sub-optimal way.

Intriguingly, we observed significant activation of a *CDC25A* promoter reporter lacking these FOXM1 binding sites. This *CDC25A* promoter reporter contained two E2F binding sites that are known to be critical for *CDC25A* transcriptional activity [30], [32] (Fig. 3D). E2F1, E2F2, and E2F3 transcriptionally activate targets essential to the G1/S transition and bind the *CDC25A* promoter [44]. We show that FOXM1 appears to indirectly activate *CDC25A* through these E2F sites (Fig. 3E). When both E2F binding sites in the *CDC25A* promoter were mutated, there was a significant decline in FOXM1-mediated activation. FOXM1 regulates the transcription of E2F genes such as E2F1, E2F2 but not E2F3, as evidenced by the fact that the transcription levels of E2F genes significantly decreased with the knockdown of FOXM1 (See Fig. S3). These data strongly support a model in which FOXM1 regulates CDC25A transcription and G1/S cell cycle transition through direct binding of FOXM1 consensus sites and through indirect activation of E2F pathways.

FOXM1 transcriptional activity, and its subsequent effect on the cell cycle, is closely tied to its phosphorylation status [45]. FOXM1 proteins are present in cells at the G1/S boundary, and, while transcriptionally active, are hypophosphorylated. The phosphorylation status of FOXM1 increases through S phase and the G2/M transition, reaching its peak hyperphosphorylated form at mitosis [3]. With the increase in phosphorylation, there is an increase in transcriptional activity. Upon exit from mitosis, cells are dephosphorylated and transcriptional activity wanes [45]. FOXM1 phosphorylation is activated by numerous kinases and kinase pathways throughout cell cycle, including Raf/MEK/MAPK pathways [8], PLK1 [13], and cyclin-dependent kinases like CDK1 and CDK2 [7], [45]–[47]. CDKs form complexes with specific cyclins at different phases of cell cycle in order to promote cell cycle progression [34]. FOXM1 preferentially associates with CDK2/cyclin E complexes during G1 and S phases of the cell cycle [48], [49] and preferentially associates with CDK1/cyclin B complexes in the G2 phase [7]. CDK-cyclin complexes associate with FOXM1 proteins at specific cyclin-docking LXL motifs and promote the phosphorylation of FOXM1 [7]. Under certain circumstances, CDK1 can substitute for CDK2 function during the G1/S transition [50]. It was reported that CDC25B protein activates CDK1/cyclin B complexes by dephosphorylation, allowing the CDK1/cyclin B complex to maintain phosphorylation of FOXM1, while activated FOXM1 allows transcription regulation of genes required to mediate the cell cycle transition (33). It is well known that CDC25A directly binds, dephosphorylates and activates CDK/cyclin through the removal of the inhibitory phosphates [34]. In the current study, CDK1, but not CDK2 mediated the CDC25A-enhanced FOXM1 transcriptional activity.

CDC25A protein directly interacts with FOXM1 protein, and phosphorylation of Thr 600 and Thr 611 residues enhanced this interaction (Fig. 6). The protein interaction of FOXM1 and CDC25A is dependent upon the phosphatase activity of CDC25A. Inactivation of CDC25A phosphatase significantly decreased the interaction of the FOXM1 and CDC25A proteins. This suggests that the CDK1/cyclin complex activated by CDC25A phosphatase is required for the protein interaction. Furthermore, inactivation of CDC25A phosphatase significantly decreased CDC25A-enhanced FOXM1 transcriptional activity (Fig. 7). The direct protein-protein interaction of CDC25A and FOXM1 indicates that FOXM1 may be a novel substrate of CDC25A phosphatase. However, we found no evidence that FOXM1 is a substrate of CDC25A-mediated dephosphorylation. Addition of CDC25A increased FOXM1 transcriptional activity. A more complicated signal transduction network likely exists between CDC25A, CDK1 and FOXM1, because FOXM1 protein interacts with many other cell cycle regulatory proteins, including CDK1, cyclin B1, CDK2, cyclin E, CDC25B, and RB [7]. The present report reveals a novel signal transduction feedback loop between CDC25A and FOXM1. CDC25A and CDK1 proteins interact, and CDK1 phosphorylates Thr 600 and Thr 611 of FOXM1. Because phosphorylation of Thr 600 and Thr 611 is required for relief of the inhibitory function of the N-terminal repressor domain during G2 phase [9], our results infer a novel mechanism by which CDC25A activates FOXM1 by relieving autorepression by the N-terminal domain.

In summary, our data provide evidence that FOXM1 directly regulates *CDC25A* gene transcription through protein-DNA binding to the promoter and indirectly through E2F-dependent mechanism. Conversely, FOXM1 transcriptional activity is synergistically enhanced when co-expressed with CDC25A. This increase is dependent upon CDK1 phosphorylation of FOXM1 protein at T600 and T611 residues. FOXM1 and CDC25A protein interact via the C-terminus of FOXM1. The phosphorylation of Thr 600 and Thr 611 residues enhanced the interaction, and the interaction is dependent upon CDC25A phosphatase activity. The evidence provided in this work sheds important new insights into the underlying mechanisms by which FOXM1, through its associations with CDC25A, regulates cell cycle progression.

## Supporting Information

Figure S1 FOXM1 inhibitor siomycin A decreased the transcription of CDC25A. U2OS cells were treated with siomycin A at 0.5 µM and 1 µM for 24 hours. The RNA was extracted and the gene transcription was tested by the qRT-PCR. Data are presented as the mean ± SD (N = 5). *, *P*<0.05; **, *P*≤0.01; and ***, *P*≤0.001.
(TIF)

Figure S2 The sequence of CDC25A promoter. The consensus binding sites for FOXM1 or E2F are highlighted with the boxes.
(TIF)

Figure S3 FOXM1 inhibition by the siomycin A down-regulated E2F1 and E2F2. U2OS cells were treated with siomycin A at 0.5 µM and 1 µM for 24 hours. The RNA was extracted and the gene transcription was tested by qRT-PCR. Data are presented as the mean ± SD (N = 5). *, *P*<0.05; **, *P*≤0.01.
(TIF)

Figure S4 CDK1 inhibitor but not CDK2 inhibitor blocked CDC25A-activated FOXM1 transcriptional activity. (A) Inhibition of CDK1 blocked CDC25A-mediated FOXM1 transcriptional activity. U2OS cells were co-transfected with pACT-CDC25A and pACT-FOXM1 and pGL3-6XFOXM1-Luc plasmids for 48 hours, and pRL-SV40 was used as the control. The cells were treated with CDK1 inhibitor (3-(2-Chloro-3-indolylmethylene)-1,3-dihydroindol-2-one) for additional 24 hours. Data were normalized to *Renilla* luciferase activities and are presented as the mean ± SD (N = 3). (A) Inhibition of CDK2 (CDK2 inhibitor II) did not block CDC25A-mediated FOXM1 transcriptional activity. U2OS cells were co-transfected with pACT-CDC25A and pACT-FOXM1 and pGL3-6XFOXM1-Luc plasmids for 48 hours, and pRL-SV40 was used as the control. The cells were treated with CDK2 inhibitor for an additional 24 hours. Data were normalized to *Renilla* luciferase activities and are presented as the mean ± SD (N = 3). *, *P*<0.05; **, *P*≤0.01; and ***, *P*≤0.001.
(TIF)

Figure S5 Efficacy of siRNA-mediated knockdown of CDK1, CDK2, CDK4, and CDK6. U2OS cells were co-transfected with pACT-CDC25A, pACT-FOXM1 and pGL3-6XFOXM1-Luc plasmids, or pACT-FOXM1 and pACT-CDC25A together with siRNAs for CDK1, CDK2, CDK4 or CDK6 or control siRNA, and pRL-SV40 was used as the control. Forty-eight hours after transfection, the cells were lysed and firefly and *Renilla* luciferase activities were tested. Lysates were also used to test the efficiency of siRNA-mediated knockdown of CDKs via western blot analysis.
(TIF)

Table S1 List of Primers used to generate the constructs.
(DOC)
