# Supplementary figures and images for: Novel Interactions between FOXM1 and CDC25A Regulate the Cell Cycle
Source: PLoS One. 2012 Dec 11;7(12):e51277. doi: 10.1371/journal.pone.0051277 (PMC3519786; doi:10.1371/journal.pone.0051277)

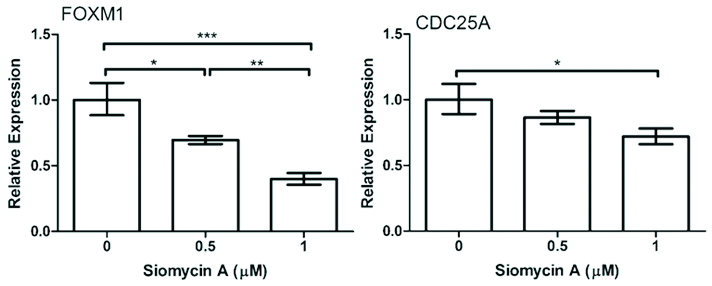

Supplement: Figure S1 — FOXM1 inhibitor siomycin A decreased the transcription of CDC25A. U2OS cells were treated with siomycin A at 0.5 µM and 1 µM for 24 hours. The RNA was extracted and the gene transcription was tested by the qRT-PCR. Data are presented as the mean ± SD (N = 5). *, P<0.05; **, P≤0.01; and ***, P≤0.001. (TIF) [file pone.0051277.s001.tif]

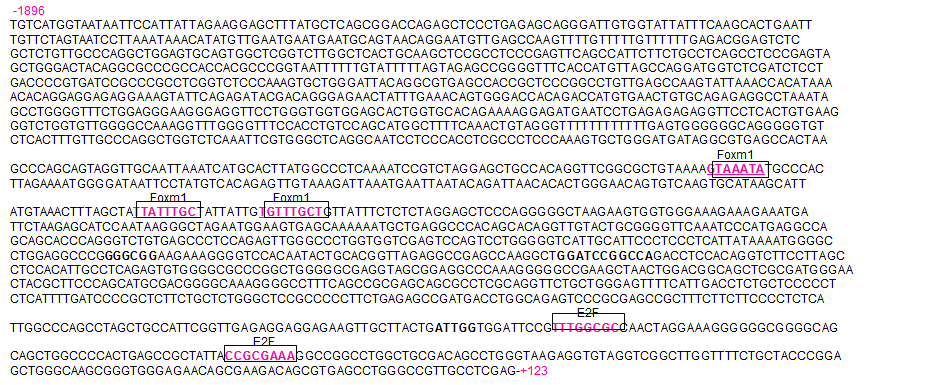

Supplement: Figure S2 — The sequence of CDC25A promoter. The consensus binding sites for FOXM1 or E2F are highlighted with the boxes. (TIF) [file pone.0051277.s002.tif]

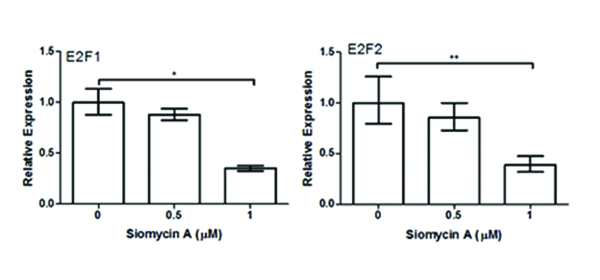

Supplement: Figure S3 — FOXM1 inhibition by the siomycin A down-regulated E2F1 and E2F2. U2OS cells were treated with siomycin A at 0.5 µM and 1 µM for 24 hours. The RNA was extracted and the gene transcription was tested by qRT-PCR. Data are presented as the mean ± SD (N = 5). *, P<0.05; **, P≤0.01. (TIF) [file pone.0051277.s003.tif]

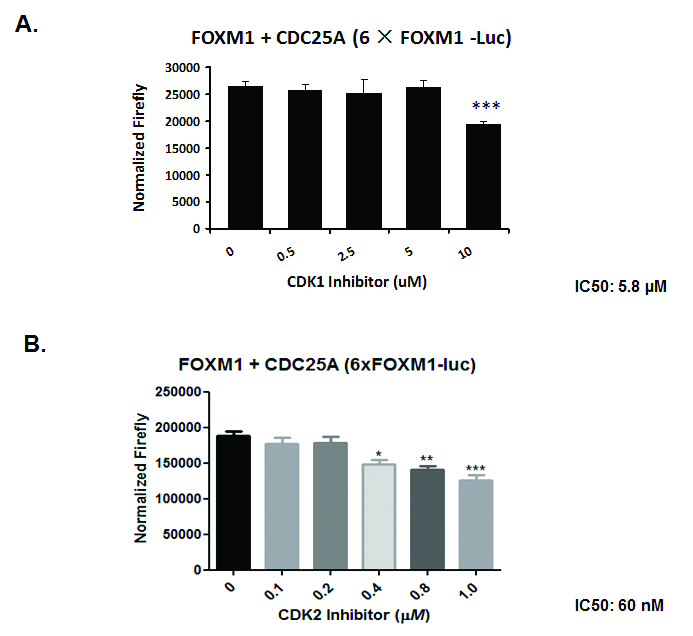

Supplement: Figure S4 — CDK1 inhibitor but not CDK2 inhibitor blocked CDC25A-activated FOXM1 transcriptional activity. (A) Inhibition of CDK1 blocked CDC25A-mediated FOXM1 transcriptional activity. U2OS cells were co-transfected with pACT-CDC25A and pACT-FOXM1 and pGL3-6XFOXM1-Luc plasmids for 48 hours, and pRL-SV40 was used as the control. The cells were treated with CDK1 inhibitor (3-(2-Chloro-3-indolylmethylene)-1,3-dihydroindol-2-one) for additional 24 hours. Data were normalized to Renilla luciferase activities and are presented as the mean ± SD (N = 3). (A) Inhibition of CDK2 (CDK2 inhibitor II) did not block CDC25A-mediated FOXM1 transcriptional activity. U2OS cells were co-transfected with pACT-CDC25A and pACT-FOXM1 and pGL3-6XFOXM1-Luc plasmids for 48 hours, and pRL-SV40 was used as the control. The cells were treated with CDK2 inhibitor for an additional 24 hours. Data were normalized to Renilla luciferase activities and are presented as the mean ± SD (N = 3). *, P<0.05; **, P≤0.01; and ***, P≤0.001. (TIF) [file pone.0051277.s004.tif]

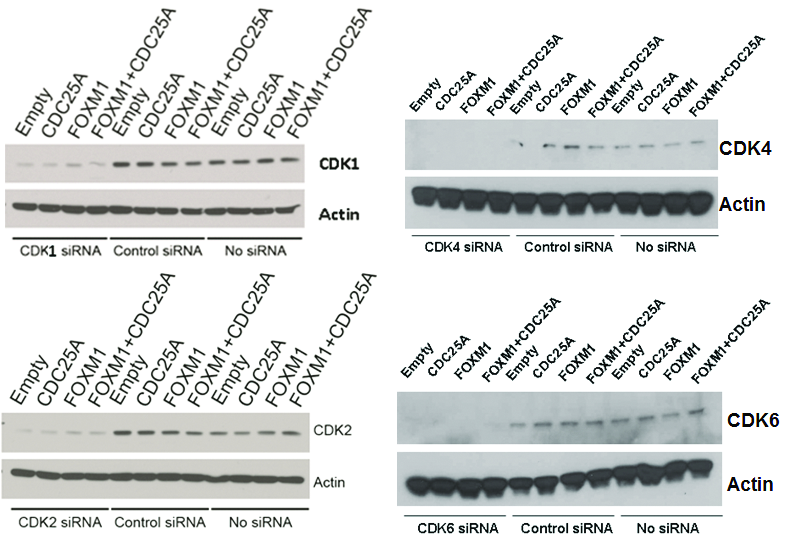

Supplement: Figure S5 — Efficacy of siRNA-mediated knockdown of CDK1, CDK2, CDK4, and CDK6. U2OS cells were co-transfected with pACT-CDC25A, pACT-FOXM1 and pGL3-6XFOXM1-Luc plasmids, or pACT-FOXM1 and pACT-CDC25A together with siRNAs for CDK1, CDK2, CDK4 or CDK6 or control siRNA, and pRL-SV40 was used as the control. Forty-eight hours after transfection, the cells were lysed and firefly and Renilla luciferase activities were tested. Lysates were also used to test the efficiency of siRNA-mediated knockdown of CDKs via western blot analysis. (TIF) [file pone.0051277.s005.tif]
