# Supplementary material for: Novel Interactions between FOXM1 and CDC25A Regulate the Cell Cycle
Source: PLoS One. 2012 Dec 11;7(12):e51277. doi: 10.1371/journal.pone.0051277 (PMC3519786; doi:10.1371/journal.pone.0051277)
Supplement: Table S1 — List of Primers used to generate the constructs. (DOC) [file pone.0051277.s006.doc]

**Supplementary Table (List of Primers used to generate the constructs)**

| CDC25A promoter | Forward | Reverse |
| --- | --- | --- |
| Full length | ATAGCCAGATCT TAGAAGGAGCTTTATGCTCAGCGG | ATAGCCAAGCTT AAACACAAACACGACTCCGCGGTT |
| -1962 through -549 | ATAGCCAGATCT TAGAAGGAGCTTTATGCTCAGCGG | ATAGCCAAGCTT CGGAATCCACCAATCAGTAAG |
| -548 through +34 | a generous gift from Dr. Daniel DiMaio Yale University |  |
| -1302 through -1078 | ATAGCCAGATCT CGTCTAGGAGCTGCCACAGGTTC | ATAGCCAAGCTT CTTCTTAGCCCCCTGGGAGCTCC |
|  | | |
| CDC25A promoter mutagenesis | Sense | Antisense |
| FOXM1 site 1 | GGCGCTGTAAAAGTAGCTATGCCCACTTA | TAAGTGGGCATAGCTACTTTTACAGCGCC |
| FOXM1 site 2 | AAACTTTAGCTATTAGCTGCTATTATTGTG | CACAATAATAGCAGCTAATAGCTAAAGTTT |
| FOXM1 site 3 | TGCTATTATTGTGTGCGCTGTTATTTCTCT | AGAGAAATAACAGCGCACACAATAATAGCA |
| E2F site 1 | ACTGATTGGTGGATTCCGTTTGAAGCCAACTAGGAAAGGG | CCCTTTCCTAGTTGGCTTCAAACGGAATCCACCAATCAGT |
| E2F site 2 | CCCACTGAGCCGCTATTACCTTGAAAGGCCGGCCTGGCTGCGAC | GTCGCAGCCAGGCCGGCCTTTCAAGGTAATAGCGGCTCA  GTGGG |
|  | | |
| CDC25A C431S Mutagenesis | Sense | Antisense |
|  | TGTCATTGTTGTGTTTCACAGCGAGTTTTCTTCTGAGAG | CTCTCAGAAGAAAACTCGCTGTGAAACACAACAATGACA |
|  | | |
| FOXM1 Deletion Constructs (pACT/pBIND) | Forward | Reverse |
| Full length FOXM1 | ACGACG GGATCCGT AAAACTA GCCCCCGTCG GCC | ACG ACG TCTAGA CTACTGTAGCTCAGGAATAAAC |
| N-term | ACGACG GGATCCGT AAAACTA GCCCCCGTCG GCC | ACG ACG TCTAGA CTA CTCAGACACAGAGTTCTGCC |
| ΔC | ACGACG GGATCCGT AAAACTA GCCCCCGTCG GCC | ACG ACG TCTAGA CTA TGGGTCCAGTGGCTTAAA |
| C-term | ACG ACG GGATCC GT GGGTCTCCACAATTGCCCGAG | ACG ACG TCTAGA CTACTGTAGCTCAGGAATAAAC |
| ΔN | ACG ACG GGATCC GT CGGCCACCCTACTCTTACATG | ACG ACG TCTAGA CTACTGTAGCTCAGGAATAAAC |
| WHD | ACG ACG GGATCC GT CGGCCACCCTACTCTTACATG | ACG ACG TCTAGA CTA TGGGTCCAGTGGCTTAAA |
| ΔWHD |  |  |
| step 1 (2 PCRs) |  |  |
| 5' end | ACGACG GGATCCGT AAAACTA GCCCCCGTCG GCC | CTCGGGCAATTGTGGAGACCCCTCAGACACAGAGTTCT  GCCA |
| 3' end | TGGCAGAACTCTGTGTCTGAG GGGTCTCCACAATTGCCCGAG | ACG ACG TCTAGA CTACTGTAGCTCAGGAATAAAC |
| step 2 (1 PCR) | ACGACG GGATCCGT AAAACTA GCCCCCGTCG GCC | ACG ACG TCTAGA CTACTGTAGCTCAGGAATAAAC |
|  | | |
| FOXM1-3xFLAG | Forward | Reverse |
| p3xFLAG-CMV-14-FOXM1 | ACGACGAAGCTTCACCATGAAAACTAGCCCCCGTCGG | ACGACGTCTAGACTGTAGCTCAGGAATAAAC |
|  | | |
| CDC25A Constructs | Forward | Reverse |
| pACT | ACG ACG GGATCC GT GAACTGG GCCCGGAGCC C | ACG ACG GCGGCCGC TCAGAGCTTCTTCAGACGACT |
| pCMV3Tag9 | ACG ACG GGATCC ACC ATGGAACTGG GCCCGGAGCC | ACG ACG CTCGAG GAGCTTCTTCAGACGACTGT |
